# Development and Evaluation of SNOMED CT Automated Mapping Tool: Advancing Terminology Standardization and Semantic Interoperability

**DOI:** 10.2196/82670

**Published:** 2026-03-09

**Authors:** Youngsun Park, Hannah Kang, Jiwon Kim, Soo-Yong Shin, Dosang Cho, Sang Youl Rhee, Hong Seok Park, Kyung-Jae Lee, Sungchul Bae

**Affiliations:** 1 Kakao Healthcare Corp Seongnam-si Republic of Korea; 2 Department of Neurosurgery Ewha Womans University Medical Center Seoul Republic of Korea; 3 Center for Digital Health Kyung Hee University Seoul Republic of Korea; 4 Department of Endocrinology and Metabolism Kyung Hee University College of Medicine Seoul Republic of Korea; 5 Department of Urology Korea University College of Medicine Seoul Republic of Korea; 6 Department of Orthopedic Surgery Keimyung University Dongsan Hospital Daegu Republic of Korea

**Keywords:** large language model, health information interoperability, systematized nomenclature of medicine, terminology, standardization

## Abstract

**Background:**

Effective secondary use of healthcare data is hindered by fragmentation and a lack of semantic interoperability due to heterogeneous local terminologies. Standardizing clinical terms using SNOMED CT (Systematized Nomenclature of Medicine Clinical Terms) is essential but remains a manual, labor-intensive, and inconsistent process, especially across multiple institutions. Automated, scalable solutions are needed to support reliable mapping and new concept authoring for large-scale research.

**Objective:**

We aimed to develop a large language model (LLM)-assisted tool that streamlines SNOMED CT terminology mapping and concept authoring, which enables seamless, standardized data integration across multi-institutional clinical datasets.

**Methods:**

The mapping pipeline included preprocessing local terms, syntactic and LLM-based vector similarity mapping, and iterative enrichment based on validated results. Translation and semantic representation used GPT-4o (OpenAI). New concepts were authored through a structured postcoordination process, and both the efficiency and quality of authoring (including duplicate rate and Machine Readable Concept Model validation violations) were quantitatively evaluated. Performance was evaluated using diagnostic and surgical procedural terms from 4 major hospital networks (9 university hospitals) in South Korea, with additional usability feedback gathered from clinical terminologists.

**Results:**

Using reference terms, top-5 accuracy for diagnostic mapping reached 98.7%, 89.7%, 98.5%, and 92.8% across the 4 institutions and 99.2%, 82.6%, 98.7%, and 84.7% for surgical procedural mapping. Implementation of the tool reduced manual mapping rates by 30% and overall manual workload by up to 90%. The proposed tool reduced average mapping and new concept creation time by approximately 75%, while decreasing the final mapping table processing time by 90%. New concept authoring errors also decreased, with duplicate concepts reduced by 83% and modeling rule violations by 72%.

**Conclusions:**

This study developed and validated an automated, LLM-assisted SNOMED CT mapping tool that significantly improved efficiency, mapping accuracy, and new concept quality. Limitations include technical integration challenges and dependency on translation quality. Future directions involve leveraging SNOMED CT’s ontology structure and knowledge graphs, enhancing sustainability through ongoing maintenance and quality assurance, and further advancing new concept authoring with automated Machine Readable Concept Model rule enforcement and inactivation processes to achieve robust and scalable terminology standardization.

## Introduction

### Background

The secondary use of healthcare data holds tremendous promise for advancing medical research and improving patient outcomes [1,2]. However, several critical barriers—such as data fragmentation, the absence of standardized medical terminologies, and semantic interoperability—continue to impede its effective reuse, particularly across multiple institutions [3]. Data generated in different health care settings are often siloed and recorded using heterogeneous systems and local coding practices, resulting in substantial misinterpretation and incomplete integration [4]. Furthermore, these challenges are compounded by the unstructured nature of much clinical data and stringent privacy and security requirements [5,6].

Achieving semantic interoperability—the ability of systems and organizations to exchange data with unambiguous, shared meaning—is essential to overcoming these challenges and unlocking the true value of multi-institutional clinical research [7,8]. At the core of semantic interoperability is terminology standardization, which enables consistent representation and interpretation of clinical concepts across diverse sources [9]. Without such standardization, variations in medical terminology across institutions can lead to inconsistencies, miscommunications, and errors that ultimately undermine the quality and reliability of biomedical research [10]. This challenge has driven international efforts, such as the World Health Organization Digital Health Interoperability Frameworks and Centers for Disease Control and Prevention National Electronic Disease Surveillance System, to prioritize standardized terminologies and interoperable health data infrastructure on a global scale [11,12].

In line with these global initiatives and the growing demands for healthcare data interoperability, Kakao Healthcare has developed the Healthcare Data Research Suite (HRS)—a platform supporting multi-institutional research via standardized data models, advanced artificial intelligence (AI) tools, and robust data privacy features. Notably, HRS places terminology standardization at its core, reflecting both the operational realities and strategic priorities identified by international guidelines [11,12]. By integrating domain-specific standard terminologies, HRS aims to bridge the gaps between fragmented datasets and promote data sharing for research.

To realize this goal, HRS adopts SNOMED CT (Systematized Nomenclature of Medicine Clinical Terms) as its primary terminology. SNOMED CT is recognized worldwide as one of the most comprehensive clinical terminology systems, encompassing a wide range of domains including disorders and procedures [13,14]. Its hierarchical structure, postcoordination capabilities, and rich attribute relationships support the precise representation of clinical concepts and sophisticated querying across large datasets [15]. Despite these advantages, mapping local clinical terms to SNOMED CT remains a significant bottleneck: the process is labor-intensive, demands expertise, and often results in inconsistent mappings both within and across institutions [16].

Recent studies have explored automated SNOMED CT mapping using various AI approaches. Davidson et al [17], 2025, benchmarked SNOMED CT entity linking models by organizing a challenge using annotated clinical notes. Gehrmann et al [18], 2025, evaluated how variable formulation impacts MedCAT’s SNOMED CT mapping performance. Kulyabin et al [19], 2024, developed and tested SNOBERT, a BERT-based entity linking model for clinical notes. While these solutions have made meaningful progress in the field, the requirements of multi-institutional research in our context further necessitated several key capabilities: (1) consistent mapping of diverse local terms, both within and across institutions; (2) management of bilingual language inputs, such as the simultaneous use of Korean and English; (3) incorporation of domain-specific curation rules; and (4) the ability to author new SNOMED CT concepts, to achieve accurate mapping that preserves the detailed meaning of source terms.

To meet these requirements and enable reliable standardization across multi-institutional studies, we have developed a large language model (LLM)–assisted terminology standardization tool that integrates automated SNOMED CT mapping and postcoordination in a unified workflow. This tool is specifically designed to overcome the challenges of mapping local terms from heterogeneous sources, handle multilingual data, and facilitate the flexible authoring of new concepts as required for large-scale clinical research.

### Objectives

The objective of this work was to create an LLM-assisted tool that enhances terminology standardization for multi-institutional research by seamlessly integrating automated SNOMED CT mapping and postcoordination within a single solution.

## Methods

### Requirement Collection

The participants involved in user requirements collection included 10 experienced clinical terminologists and 3 data engineers. Participants were asked to provide feedback on essential features through an open-ended questionnaire.

### System Architecture and Implementation

**Figure 1:**
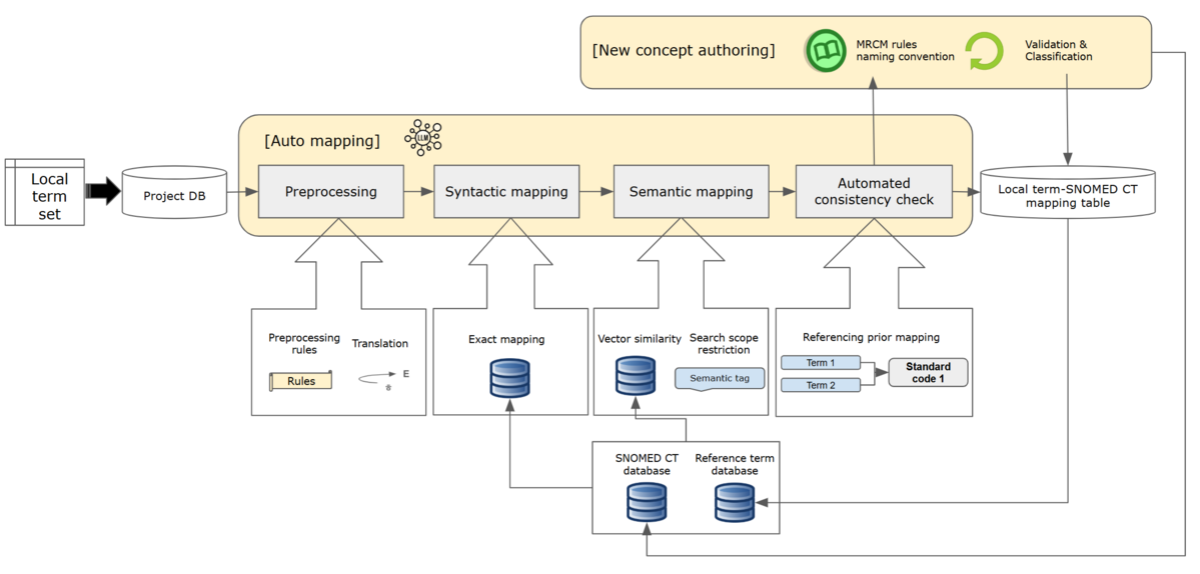
Overall workflow of the LLM-assisted terminology standardization tool. LLM: large language model; MRCM: Machine Readable Concept Model; SNOMED CT: Systematized Nomenclature of Medicine Clinical Terms.

The overall process of automatic mapping is illustrated in Figure 1. The workflow consists of 2 main processes: auto-mapping and new concept authoring. The final mappings are progressively incorporated into the local term-SNOMED CT mapping database and integrated into the custom reference term database, which includes the previous mapping results of local codes.

#### Auto-Mapping Method

##### Overview

**Figure 2:**
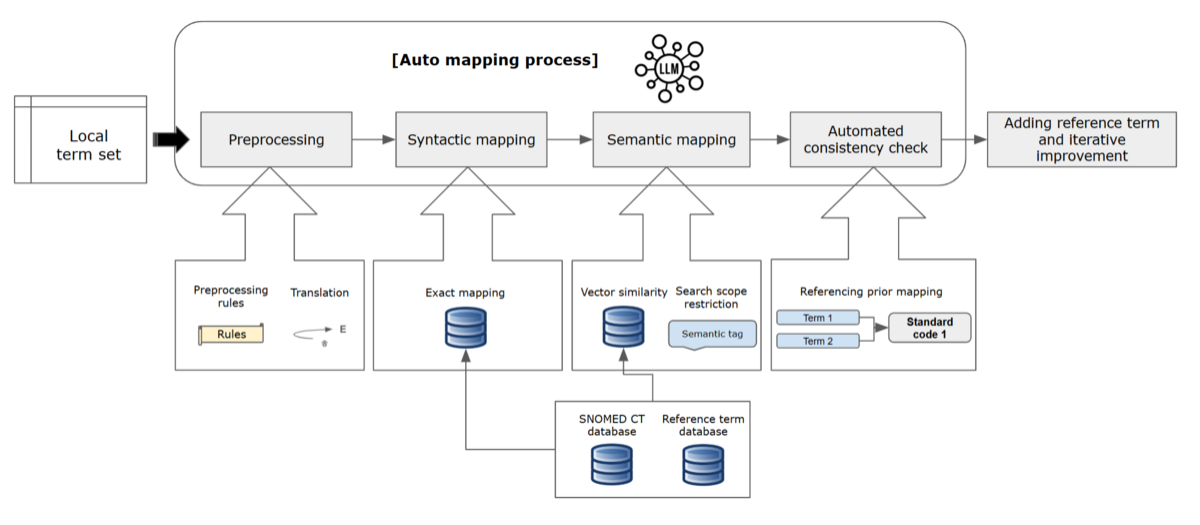
Overview of the LLM-assisted automated SNOMED CT mapping process for multi-institutional standardization. LLM: large language model; SNOMED CT: Systematized Nomenclature of Medicine Clinical Terms.

The automated mapping process, as illustrated in Figure 2, begins with receiving a local term set and sequentially integrates four distinct components: (1) data preprocessing, (2) syntactic mapping, (3) semantic mapping, and (4) automated consistency check.

##### Data Preprocessing

Data preprocessing consists of three main steps: (1) removing unnecessary symbols, (2) expanding abbreviations to full terms, and (3) translating non-English terms such as Korean into English with LLM tools (eg, GPT-4o (OpenAI) or Gemini 2.0 Flash (Google)). In the first step, unnecessary symbols are removed based on professional review. Second, abbreviations are converted to their full forms through manual curation, as context-dependent abbreviations such as “MS” can have multiple meanings (“multiple sclerosis” in neurology or “mitral stenosis” in cardiology); automatic expansion is therefore avoided. Third, any local terms in Korean are translated to English using LLMs. Although GPT-4o was used in this study, the system is designed to flexibly substitute other models (eg, Gemini 2.0 Flash) to accommodate institutional IT or cloud service restrictions.

Furthermore, to maximize long-term accuracy, we implemented a postmapping rule-based refinement mechanism. This system analyzes the automated mapping output and removes nonessential terms or extraneous symbols that do not affect similarity scores. This quality assurance step ensures that these refined terms are immediately incorporated into the custom reference term database for iterative use, thereby boosting subsequent mapping performance.

To enhance the reproducibility and institutional specificity, the system allows each participating institution to define and apply custom rule-based preprocessing before automated mapping. These user-defined rules are automatically applied within the mapping pipeline, allowing each site to enforce its own normalization standards as needed.

##### Syntactic Mapping

Following the data preprocessing steps, the syntactic mapping component performs exact mapping by identifying standard codes that are textually identical to the preprocessed local term. To achieve this, the component prioritizes a search that is restricted within the SNOMED CT description table. In addition, the search leverages custom reference terms, which function similarly to the SNOMED CT description and were developed based on previously confirmed mapping results. If a textual identity between the normalized local term and one of the entries in the reference corpus is found, it is recommended to the user as an exact match.

##### Semantic Mapping

The semantic mapping component is used when the syntactic mapping component fails to find an exact match, using vector similarity search techniques to automatically map local terms to standard codes. This process leverages LLM-based embedding models (such as the text-embedding-3-small model for OpenAI deployments) to encode all reference terms, which include SNOMED CT descriptions and previously mapped local terms. These reference embedding results are stored in the Faiss vector database [20].

To ensure the efficiency and domain relevance of semantic search, the component applies a crucial constraint developed from empirical insights. First, the search is restricted to the SNOMED CT description table and custom reference terms validated through previous mappings. This prioritizes exact and highly relevant matches within established terminology sources. Second, the results are further refined by limiting the search scope to specific semantic tags and datasets relevant to each domain. For instance, the imaging and functional test domain is limited to “Procedure” and “Regime/Therapy” semantic tags; the diagnosis domain exclusively uses “Disorder,” “Finding,” “Situation,” “Event,” and “Person” tags. Additionally, certain cases restricted searches to descendants of specific concepts, allowing for targeted searches within specific datasets. For example, when mapping datasets for culture medium, searches were limited not only to “substance” but specifically to descendants of the concept 421955000 |Culture medium (substance)|, enhancing both speed and accuracy.

##### Automated Consistency Check

The automated consistency check component serves as the final stage in the automated mapping process. Its primary function is to ensure consistency and adherence to established mapping principles across all mapping candidates generated by the preceding components. This component achieves its goal primarily through referencing a prior mapping rule. This rule is specifically designed to maintain consistency across terms. When duplicate source terms are present, a validated mapping for 1 instance is automatically applied to all others. This rule is applied across different institutions, increasing efficiency and ensuring uniformity, except when the datasets belong to different domains. A successful automated consistency check leads directly to the final system step: adding reference terms and iterative improvement enriching the knowledge base for future mapping runs.

#### New Concept Authoring Method

**Figure 3:**
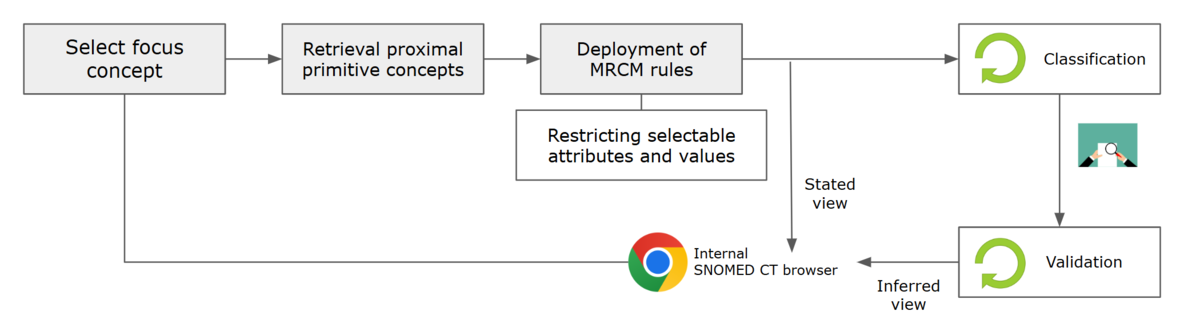
New concept authoring process using postcoordination within the LLM-assisted terminology standardization tool. LLM: large language model; MRCM: Machine Readable Concept Model; SNOMED CT: Systematized Nomenclature of Medicine Clinical Terms.

Our new concept authoring procedure closely follows the principles and steps outlined in the SNOMED CT Editorial Guide [21]. Subsequently, Figure 3 illustrates the process of the authoring of new concepts through postcoordination. The process consists of four steps: (1) the selection of a focus concept, supported by proximal primitive concept retrieval; (2) concept modeling performed in alignment with Machine Readable Concept Model (MRCM) rules [22], followed by terming; (3) classification and validation; and (4) integration with the mapping table.

In the current version of our tool, we have operationalized 2 core rules from the editorial guide: the proximal primitive modeling principle and enforcement of MRCM rules. Other elements of the editorial guidance, such as cardinality, concept inactivation workflow, and modeling templates, are not yet implemented and are under consideration for future development.

The first implemented editorial rule is proximal primitive modeling. Adhering to this principle is recommended when authoring new concepts in SNOMED CT [23]. The principle of proximal primitive modeling is vital for ensuring that classifiers accurately infer the most relevant proximal supertypes, which enhances precise subsumption [24,25]. When a user selects a focus concept required for modeling, not only the concept ID and fully specified name (FSN) of the focus concept, but also the corresponding proximal primitive concept are retrieved via the authoring-form API (Application Programming Interface) provided by Snowstorm [26].

The second implemented rule is the automated enforcement of MRCM rules. The MRCM defines the rules within the SNOMED CT concept model in a computer-readable format, allowing for validation of concept definitions and expressions to ensure compliance [27]. We have integrated these rules into the user interface (UI) by using the Snowstorm API. The tool restricts selectable attributes based on the domain of the focus concept. Using the GET /mrcm/{branch}/domain-attributes API, the focus concept ID is passed as parent IDs to retrieve only attributes relevant to the specific domain.

For naming guidance, our tool supports authors by automatically loading the FSN of the selected focus concept—retrieved via the authoring-form API—as a terming template. This allows users to customize the FSN, ensuring that the created concept precisely reflects the intended meaning, while maintaining overall consistency with SNOMED CT naming conventions.

Classification and validation are crucial steps in authoring new SNOMED CT terms for maintaining the precision and comprehensiveness of SNOMED CT and supporting accurate cohort queries over electronic health records [28]. We used the SNOMED (Systematized Nomenclature of Medicine) Release Validation Framework and the SNOMED OWL (Web Ontology Language) Toolkit in parallel to ensure the quality and consistency of our SNOMED CT editions, generate OWL expressions, and perform classification [29,30].

Once new concepts are authored, they are systematically stored in the mapping table, which is a structured database that includes the source terminology and corresponding target SNOMED CT identifiers [31]. We managed a Kakao Healthcare edition in our database that included all SNOMED International concepts, supplemented with new concepts assigned to a unique namespace identifier of the affiliated institution.

As the SNOMED CT Editorial Guide and modeling rules are subject to periodic updates, we monitor release notes and editorial guide updates on a monthly basis. While we conduct regular follow-up reviews each month, updates are applied to our edition on an annual basis through scheduled manual audits for the new concept authoring to comply with recent editorial changes.

#### System Implementation

To support the key functionalities of automatic mapping and new concept authoring, the system was implemented as 2 main components: a web application and an automatic mapping server. The web application, developed using Next.js (version 14; Vercel) and TypeScript (version 5; Microsoft), serves as the central platform that integrates various operations such as project management, data submission, mapping review, and concept authoring. React Query (version 5.28.14; TanStack) is used for managing asynchronous data interactions, and Tailwind CSS (version 3.3.0; Tailwind Labs) is used for styling. The application communicates with the backend services and the database to manage workflows and interface with the automatic mapping server. The automatic mapping server, built in Python (version 3.10; Python Software Foundation), performs the core mapping logic, including preprocessing of local terms, translation using LLM, and vector-based similarity search using FAISS (faiss-cpu version 1.9.0.post1; Meta AI Research). The server generates mapping results and stores them in a PostgreSQL (version 14.12; PostgreSQL Global Development Group) database. FastAPI (version 0.110.3; FastAPI framework) is used to serve the backend API.

### Performance Evaluation

#### Method for Evaluating Accuracy

##### Dataset

The dataset for evaluating performance was collected from 4 hospital networks (a total of 9 university hospitals) in South Korea: Ewha Womans University Medical Center (EUMC) with 2 branches, Korea University Medical Center (KUMC) with 3 branches, DongSan Medical Center (DSMC) with 2 branches, and KyungHee University Medical Center (KHMC) with 2 branches. The selection of diagnostic and surgical procedure terms is strategically made due to their clear and distinct alignment with specific SNOMED CT semantic tags, such as “Disorder” and “Procedure,” which facilitates precise mapping. Furthermore, these codes encompass nearly all medical specialties, ensuring the heterogeneity of local medical practice in South Korea, thereby providing a robust dataset for evaluating the mapping tool’s performance. EUMC contributed 24,554 diagnostic and 8053 surgical procedure codes, KUMC provided 44,646 and 13,734, respectively, DSMC supplied 24,146 and 9432, while KHMC offered 27,842 diagnostic codes and 20,759 surgical procedure codes.

##### Preprocessing Rules

All performance evaluations were conducted using a common, standardized set of preprocessing rules applied uniformly across all 4 participating hospital networks (EUMC, KUMC, DSMC, and KHMC). The system’s “user-defined rules” functionality, while supported for production deployment, was intentionally not used during the formal testing in this study. This ensured that the comparison of mapping accuracy reflected the system’s performance under standardized, reproducible conditions.

##### LLM Configuration

All translation and concept generation tasks in this study were performed using GPT-4o (OpenAI, 2024) and its corresponding text-embedding-3-small embedding model for vector-based similarity mapping. The selection of GPT-4o was fixed across all institutions to ensure methodological consistency and reproducibility. All reported accuracy, efficiency, and usability metrics are therefore based exclusively on results generated from the GPT-4o-based pipeline.

##### Accuracy Assessment Strategy

The performance was measured by comparing its outputs against manually mapped results, which have been established as the gold standard. The manual mapping results were verified for accuracy and reliability through at least 2 rounds of internal review by 5 clinical terminology experts and 1 physician, and were further refined by external review with 2 physicians.

Accuracy was evaluated using both “top-1” and “top-5” approaches. The top-1 accuracy refers to the highest similarity score of a candidate being correct. The top-5 accuracy includes having the correct mapping among the 5 candidates with the highest similarity scores. Furthermore, to better characterize the contribution of the shared knowledge base versus the AI components, we stratified the “accuracy with reference terms” metric. We specifically calculated the “exact match” rate to distinguish successful mappings achieved via syntactic reference identity lookups (memorization from partner hospitals) from those achieved via vector-based semantic similarity search.

Specifically, we used a 2-pronged approach to measure mapping accuracy. First, we assessed the baseline performance at each participating hospital—EUMC, KUMC, DSMC, and KHMC—by using only the descriptions of the SNOMED CT Kakao Healthcare edition. The SNOMED CT Kakao Healthcare edition serves as the core internal database, managing all SNOMED International concepts and supplemented with new concepts assigned a unique namespace identifier of the affiliated institution. Crucially, the baseline analysis intentionally excludes any reference terms (previously confirmed mappings) that were accumulated or validated from the other participating hospital networks.

Second, we enhanced our analysis by adding reference terms from the other participating hospitals into the mapping process. For instance, when assessing EUMC, we introduced reference terms from KUMC, DSMC, and KHMC to gauge any changes in mapping accuracy. This comparative methodology was implemented in stages to allow us to compare the tool’s performance before and after the addition of reference terms, as mapping data from other hospitals accumulated. The benefit measured in the subsequent metric, “accuracy with reference terms,” therefore represents the isolated effect of integrating this shared, cross-institutional mapping knowledge.

##### Handling of “Not Mapped” Terms

To ensure comprehensive performance accounting, we clarified the treatment of local terms designated as “not mapped” within the accuracy calculation. These “not mapped” terms were established as part of the gold standard definition by the clinical terminology review committee. Such terms often represent local entries that human experts determined lacked a valid or appropriate SNOMED CT concept (eg, referencing only body structures or materials without a corresponding procedure).

Crucially, the system was not explicitly designed to predict a “not mapped” status. Therefore, when a term is validated by experts as “not mapped” (the gold standard outcome), the system’s failure to provide any correct SNOMED CT prediction within the top-1 or top-5 candidates is inherently reflected in the overall accuracy calculation as a system prediction failure or error.

#### Method for Evaluating Efficiency

As a baseline, all processes were manually executed to assess the time required for each step. Data preprocessing time is the average time required for data cleaning and translation. New concept authoring time refers to the average time it takes for a person to author a new concept in compliance with SNOMED CT’s principles. The final processing steps of the mapping table refer to the average time taken per dataset for integrating mapping tables and verifying data integrity. This observation is based on a 6-month operational period of a clinical terminology team consisting of 6 members. During this time, detailed logs and time-tracking data were collected across all stages of the terminology mapping process.

The results from the tool-based workflows were compared with the baseline data to evaluate the improvements: (1) efficiency gains are the reduction in time for each phase of the process, expressed as a percentage improvement over baseline; and (2) resource optimization evaluates the changes in human resource consumption.

#### Quality Assessment of New Concept Authoring

Quality was evaluated pre- and posttool adoption using 2 metrics derived from the full SNOMED CT authoring dataset as of August 27, 2024. First, the proportion of duplicate concepts was calculated by dividing the number of equivalent errors identified after classification by the total number of authored concepts at each time point. Second, the number of MRCM validation violations was measured by counting rule violations across all authored concepts before and after tool implementation.

#### System Usability Evaluation by Users

The system usability evaluation was conducted with 5 mappers who had previous experience with mapping methodologies before the tool development. These participants were introduced to the main features of the newly developed tool and were asked to perform approximately 100 sample mappings. After using the tool, they were encouraged to provide feedback in an open-ended format regarding their experience with the tool.

### Ethical Considerations

This study was conducted as part of the research project “Development of an LLM-based Named Entity Recognition (NER) tool and a standardization support tool for using multicenter clinical free text.” As no human participants or identifiable personal information were involved, institutional review board (IRB) approval was not required for this study itself. However, IRB approval or exemption was obtained from each institution for project administration purposes as follows: Ewha Womans University Medical Center (SEUMC IRB 2025-02-016, approved), DongSan Medical Center (DSMC IRB 2025-01-006, approved), Korea University Medical Center Anam (KUMC IRB 2025AN0239, approved), Guro (KUMC IRB 2025GR0222, approved), Ansan (KUMC IRB 2025AS0122, approved), and KyungHee University Medical Center (KHUH IRB 2025-01-047, exempted). This study was conducted in compliance with institutional policies, the Korean Bioethics and Safety Act [32], and the Declaration of Helsinki [33]. Informed consent was not required due to the nature of the study. 

## Results

### System Development and Deployment

#### System Development Requirements

**Figure 4:**
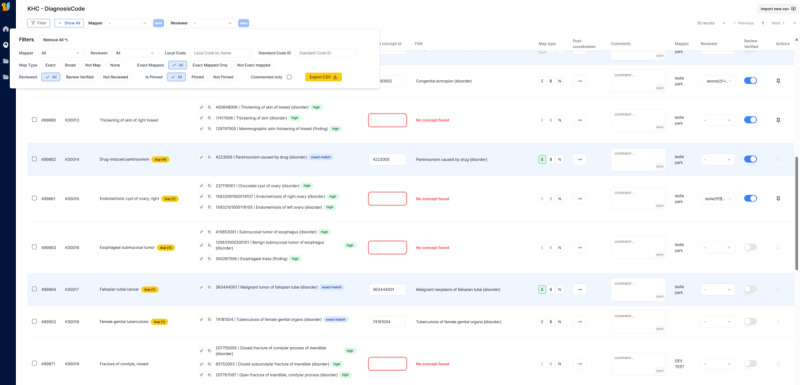
User interface of the automated mapping page within the LLM-assisted terminology standardization tool. KHC: Kakao Healthcare; LLM: large language model.

Based on the users’ survey, we found the following 11 key requirements as shown in Table 1. The UI of the auto-mapping enables users to map terms with a single click in a familiar table-based layout. Figure 4 displays the primary UI for the automated SNOMED CT mapping function. The interface is organized in a familiar table-based layout to facilitate the review and review of local diagnostic and surgical procedure terms. To maximize efficiency and minimize manual errors, the UI provides single-click mapping and automatic retrieval of FSNs immediately upon entering a standard concept ID. Furthermore, the interface supports essential collaborative functions for large-scale projects, including comprehensive filtering options and dedicated fields for review tracking and quality assurance by terminology experts.

**Table 1:** Key requirements for the LLM^a^-assisted automated SNOMED CT^b^ mapping and new concept authoring tool, defined by clinical terminologists and data engineers for multi-institutional research environments.

Category	Key requirements
Project and dataset version management	The ability to create projects and manage their versions for terminology datasets by institution and domain. Support for uploading source files and downloading mapping table result files in the computable format (CSV and JSON)
Automated mapping and terminology support	Automated mapping to eliminate the need for manual browser searches for each local term. Translation functionalities to support multilingual datasets. Management tools for mapping tables, including cross-checking mapping results.
New concept authoring and integration	Functionality to author new concepts during the mapping process. A user interface applying MRCM^c^ rules during authoring. Application of the classification of newly authored concepts. Immediate incorporation of newly authored concepts into the browser. Automatic integration of newly authored concepts into the mapping tables.
Review and quality assurance	Features for human review of newly authored concepts for naming inconsistencies and semantic modeling errors.

^a^LLM: large language model.

^b^SNOMED CT: Systematized Nomenclature of Medicine Clinical Terms.

^c^MRCM: Machine Readable Concept Model.

**Figure 5:**
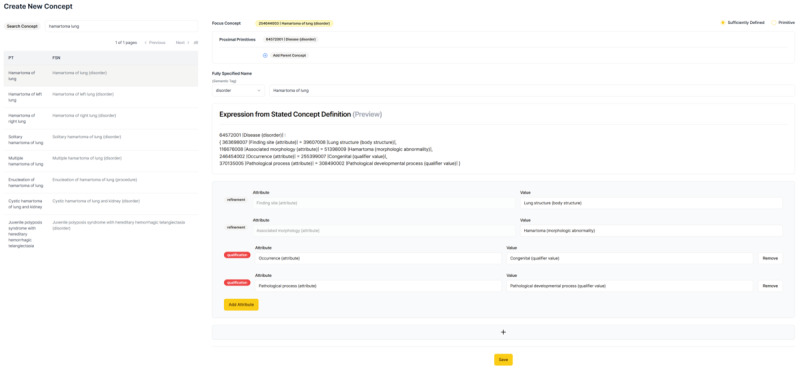
User interface of the new concept authoring page within the LLM-assisted terminology standardization tool. FSN: fully specified name; LLM: large language model; PT: preferred term.

The new concept authoring tool, as shown in Figure 5, makes it easier to author new concepts with an intuitive visual editor that supports real-time updates. In illustrating Figure 5, the concept "254644003 |Hamartoma of lung (disorder)|" is used as the focus concept. Its modeling was cloned and then customized for the illustration, as duplicating the modeling of the focus concept is often an efficient and practical method when modeling new concepts. Then, the proximal primitive concept of selected focus concept, “64572001 |Disease (disorder)|,” is automatically retrieved. Users can perform authoring within the UI through refinement or qualification methods.

#### System Deployment

The system is actively deployed for terminology standardization at the Research Alliance participating hospitals, which is organized by Kakao Healthcare. To date, it has processed terminology from a total of 6 hospital networks, encompassing 12 university hospitals and 189,455 terms. The developed tool has been actively used to standardize terms across various domains, including diagnoses, surgical procedures, chief complaints, and laboratory tests.

### System Evaluation

#### Result of Performance Evaluation

Table 2 displays the successful mappings achieved through syntactic matching (exact match lookups) using the custom reference database (baseline) vs those achieved after the iterative enrichment via shared reference terms (accuracy with reference terms) across 4 major hospital networks. The reported percentage represents the proportion of local terms that were successfully mapped by exact identity lookup. The analysis revealed that the inclusion of shared reference terms universally increased the proportion of terms resolved via exact match, reducing the reliance on vector-based search. However, the magnitude of this increase varied significantly across hospital networks.

**Table 2:** Syntactic mapping performance: exact match accuracy for diagnosis and surgical procedure domains.

Hospital networks, domain			Metric		Baseline accuracy (%)		Accuracy with reference terms (%)		Δ accuracy (%)
**EUMC^a^**									
	Diagnosis	Exact match		36.3		94.5		58.2	
	Surgical procedure	Exact match		18.5		97.6		79.1	
**KUMC^b^**									
	Diagnosis	Exact match		23.4		36.6		13.2	
	Surgical procedure	Exact match		17.7		29.8		12.1	
**DSMC^c^**									
	Diagnosis	Exact match		34.9		94.9		60	
	Surgical procedure	Exact match		19.8		96.9		77.1	
**KHMC^d^**									
	Diagnosis	Exact match		21.9		55.2		33.3	
	Surgical procedure	Exact match		12.8		28.3		15.5	

^a^EUMC: Ewha Womans University Medical Center.

^b^KUMC: Korea University Medical Center.

^c^DSMC: DongSan Medical Center.

^d^KHMC: KyungHee University Medical Center.

Notably, EUMC and DSMC exhibited a dramatic surge in exact match rates. For the diagnosis domain, exact match accuracy increased from 36.3% to 94.5% for EUMC and from 34.9% to 94.9% for DSMC. A similar trend was observed in the surgical procedure domain, where exact match rates reached 97.6% and 96.9%, respectively. In contrast, KUMC and KHMC showed more moderate increases. For the diagnosis domain, KUMC’s exact match rate improved from 23.4% to 36.6%, and KHMC’s improved from 21.9% to 55.2%. In the surgical domain, the exact match rates remained below 30% for both institutions (29.8% for KUMC and 28.3% for KHMC), though this still represented a gain of over 10% for each.

Table 3 summarizes the comparative performance evaluation of mapping accuracy for diagnostic and surgical procedure domains across 4 hospital networks. For each hospital and domain, mapping performance was measured as top-1 and top-5 accuracy, both at baseline (before incorporating reference terms) and after the inclusion of reference terms. To ensure statistical rigor, 95% CIs were calculated for all accuracy estimates. The absolute improvement in accuracy (Δ accuracy) was also calculated to quantify the benefit of leveraging shared reference mappings.

**Table 3:** Comparative performance evaluation of automated SNOMED CTa^a^ mapping accuracy for diagnostic and surgical procedure domains across 4 South Korean multi-institutional networks. All improvements were statistically significant (McNemar test, *P*<.01 for all cases). 95% CIs were calculated using the Wald method.

Hospital network, domain, not mapped rate (%), metric					Baseline accuracy (%)		Accuracy with reference terms (95% CI)		Δ accuracy^b^ (%)
**EUMC^c^**									
	**Diagnosis**								
		**0**							
			Top-1	73.4		97.4 (97.2–97.6)		24.0	
			Top-5	88.9		98.7 (98.6–98.8)		9.8	
	**Surgical procedure**								
		**0**							
			Top-1	64.9		98.5 (98.2–98.8)		33.6	
			Top-5	84.7		99.2 (99.0–99.4)		14.5	
**KUMC** ^d^									
	**Diagnosis**								
		**0.4**							
			Top-1	69.3		73.2 (72.8–73.6)		3.9	
			Top-5	87.0		89.7 (89.4–90.0)		2.7	
	**Surgical procedure**								
		**1.7**							
			Top-1	61.6		66.1 (65.3–66.9)		4.5	
			Top-5	79.0		82.6 (82.3–82.9)		3.6	
**DSMC** ^e^									
	**Diagnosis**								
		**0**							
			Top-1	72.7		97.2 (97.0–97.4)		24.5	
			Top-5	88.2		98.5 (98.3–98.7)		10.3	
	**Surgical procedure**								
		**0**							
			Top-1	66.6		97.9 (97.6–98.2)		31.3	
			Top-5	85.2		98.7 (98.5–98.9)		13.5	
**KHMC** ^f^									
	**Diagnosis**								
		**0**							
			Top-1	67.9		81.4 (80.9–81.9)		13.5	
			Top-5	85.5		92.8 (92.5–93.1)		7.3	
	**Surgical procedure**								
		**0**							
			Top-1	59.7		65.1 (64.5–65.7)		5.4	
			Top-5	81.1		84.7 (84.2–85.2)		3.6	

^a^SNOMED CT: Systematized Nomenclature of Medicine Clinical Terms.

^b^Δ accuracy: accuracy with reference terms−baseline accuracy.

^c^EUMC: Ewha Womans University Medical Center.

^d^KUMC: Korea University Medical Center.

^e^DSMC: DongSan Medical Center.

^f^KHMC: KyungHee University Medical Center.

At baseline, top-5 accuracy for diagnosis was similar across hospitals, ranging from 85.5% to 88.9%. In contrast, surgical procedures showed greater variation, with baseline top-5 accuracy from 79% to 85.2%. After reference terms were incorporated, top-5 accuracy for diagnosis increased to between 89.7% and 98.7%, and for surgical procedures it rose to between 82.6% and 99.2%. EUMC and DSMC showed the highest postreference top-5 accuracy in both domains and achieved the largest absolute improvement in surgical procedures (up to 14.5% at EUMC and 13.5% at DSMC).

For top-1 accuracy, the improvement after reference terms addition was even more pronounced, especially in surgical procedures, with EUMC reaching a 33.6% increase. Top-5 accuracy remained consistently higher than top-1 in all hospitals and domains, but the relative improvement with reference terms was greater for top-1 than for top-5. All gains in accuracy were statistically significant (McNemar test, *P*<.01). In a supplementary evaluation, the exclusion of the syntactic mapping (exact match) step resulted in negligible variations in top-5 accuracy, suggesting that the vector-based semantic search effectively identifies these identical terms.

To provide context for performance ceilings, we analyzed the prevalence of unmappable terms. The gold standard dataset included terms categorized as “not mapped,” representing local codes for which experts determined no valid SNOMED CT concept existed. These instances were treated as errors in the accuracy calculation. The prevalence of “not mapped” terms varied by institution and domain: EUMC (diagnosis: n=0, 0%; surgical procedure: n=0, 0%), KUMC (diagnosis: n=174, 0.4%; surgical procedure: n=219, 1.7%), DSMC (diagnosis: n=1, 0%; surgical procedure: n=0, 0%), and KHMC (diagnosis: n=0, 0%; surgical procedure: n=0, 0%).

#### Result of Efficiency Evaluation

**Figure 6:**
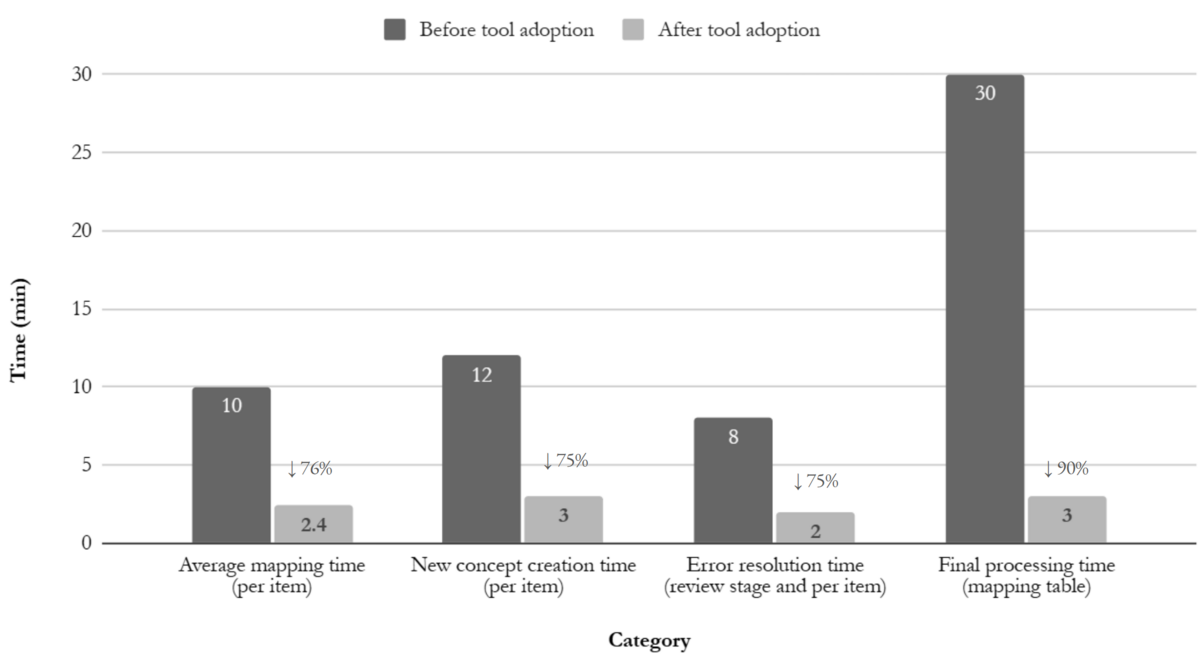
Efficiency evaluation of the SNOMED CT mapping workflow, comparing manual baseline time to performance after adopting the LLM-assisted automated tool across 4 key stages: mapping, new concept authoring, error resolution, and final processing of the mapping table. LLM: large language model; SNOMED CT: Systematized Nomenclature of Medicine Clinical Terms.

Figure 6 clearly demonstrates the efficiency evaluation based on a 6-month operational period, showing significant reductions in manual workload across 4 key stages of the SNOMED CT mapping process. The manual mapping rate decreased from 100% to 70%, indicating a 30% reduction and highlighting the tool’s effectiveness in enabling automated mapping. More broadly, the overall manual workload was significantly reduced, with marked enhancements in efficiency throughout the entire mapping process. Specifically, the average mapping time per item decreased from 10 to 2.4 minutes, representing a 76% reduction. The time required to create a new concept similarly dropped from 12 to 3 minutes (75% reduction), while the time needed to resolve errors during the review stage was reduced from 8 to 2 minutes (75% reduction). Most notably, the final processing time of the mapping table per dataset decreased from 30 to 3 minutes, achieving a 90% reduction.

#### Results of New Concept Authoring Quality Evaluation

After implementation of the tool, both classification and MRCM validation errors decreased substantially, even as the total number of authored concepts increased. The proportion of duplicate concepts dropped from 0.8% (122/15,637) with manual authoring to 0.1% (46/34,401) with the tool, representing an 83% reduction. Likewise, MRCM validation violations decreased by 72%, from 264 in the manual process to 75 with the tool-assisted workflow.

#### Result of System Usability Evaluation

Users highlighted several benefits of the system. First, the tool reduced effort during the mapping process by automatically suggesting candidate target concepts, which minimized the number of manual typing and clicking steps required. Second, users noted a significant decrease in errors, as the system eliminated mistakes that previously occurred when characters were deleted or data types were changed during manual copying and pasting of IDs or FSNs. The tool now conveniently enters the FSN when a valid ID is provided, further improving accuracy. Finally, the new system offered enhanced usability compared to the previous spreadsheet-based screens, which users found visually straining and inflexible. With the new interface, users reported a more comfortable and convenient experience.

## Discussion

### Principal Findings

This study developed an LLM-assisted SNOMED CT automatic mapping tool and evaluated it across multiple real-world hospital settings. Notably, the tool supports postcoordination, which minimizes semantic loss from source terms and leverages the strengths of SNOMED CT. In evaluation, the tool demonstrated high top-5 accuracy rates—92.6% for diagnostic concepts and 93.6% for surgical procedures. Incorporating reference mappings further increased accuracy by over 6% in both areas. Workflow efficiency was markedly enhanced, with mapping and concept authoring times reduced by approximately 75% and the proportion of cases requiring manual mapping dropping from 100% to 70%. Importantly, the tool reduced human modeling errors in new concept authoring, as evidenced by an 83% decrease in duplicate concept rate and a 72% reduction in MRCM validation violations. Error resolution and final processing of the mapping table were also significantly streamlined.

When evaluating site-specific performance, we found substantial variation across different hospital networks, especially after integrating the shared reference knowledge base. Performance in networks such as EUMC and DSMC was exceptionally high, with exact match rates exceeding 94% in diagnosis and 96% in surgical domains—likely reflecting the use of shared electronic medical record systems, which allowed most mappings to be resolved through simple identity lookups.

To analyze the actual contribution of the exact match step, we performed an internal assessment comparing mapping accuracy with and without the preliminary exact match stage. This assessment revealed that the difference in top-5 accuracy between the 2 configurations was negligible. Thus, the primary value of the exact match component lies not in enhancing end-to-end mapping quality but in workflow optimization: by quickly resolving about 30% of cases, it helps bypass computationally and financially intensive processes, contributing to pipeline efficiency and sustainability.

The accumulation and reuse of reference mappings across institutions is also central to our approach and reflects both the realities and strengths of terminology standardization initiatives in Korea; as more hospitals join and contribute to the shared repository, we anticipate continued improvements in mapping consistency and efficiency across various settings.

By contrast, KUMC and KHMC exhibited more modest improvements, as a substantial portion of their mappings could not be resolved through exact identity lookup alone. For these institutions, the shared knowledge base contributed to performance gains but was insufficient on its own; thus, many mappings still required the use of vector-based semantic similarity search. This highlights the ongoing need for advanced AI components when handling diverse or nonstandardized local terminologies that may not be well represented within a shared repository.

Further analysis of auto-mapping errors revealed 3 main sources of reduced accuracy: (1) the presence of local terms that reference only body structures or materials without an associated procedure—instances best categorized as “not mapped”; (2) terms requiring new concept creation via postcoordination—particularly in the surgical procedure domain, where postcoordination was frequently necessary; and (3) unresolved or ambiguous abbreviations. Understanding these error patterns provides important directions for future refinement of the system.

### Comparison With Prior Work

A consistent set of challenges emerges across this and prior work, including the persistent difficulty of mapping rare or institution-specific terms, handling ambiguous abbreviations, and resolving semantically unclear expressions [17-19]. Beyond algorithmic advancements, real-world clinical deployment presents unique integration hurdles, such as harmonizing diverse IT infrastructures, maintaining synchronization between terminology servers and electronic medical record systems, and managing evolving local terminologies [18]. Importantly, our study reaffirms that, regardless of automation level, human review remains indispensable—not only for establishing and maintaining gold standard mappings that underpin system performance, but also for defining and updating mapping rules to reflect evolving clinical realities [17,18].

### Limitations

First, we encountered various technical difficulties during implementation. The internal PostgreSQL database and the ElasticSearch (Elasticsearch B.V.) engine supporting SNOMED CT search (via SNOMED Snowstorm API and Browser) required frequent manual synchronization due to architectural differences. This was especially challenging since ElasticSearch lacks built-in transactional support. Accordingly, script-based data reconciliation was required to maintain consistency, but it remains to be fully automated.

Second, a significant limitation of our methodology involves the Korean-to-English translation step required for processing bilingual input data. As the accuracy of the subsequent vector similarity mapping is entirely dependent on the quality of the translated term, we acknowledge that the lack of gold-standard comparisons or error analysis for this step is a methodological limitation.

Third, translation was handled as a fully automated preprocessing step to minimize human intervention and maximize efficiency within the mapping pipeline. To control quality when using models such as GPT-4o or Gemini 2.0 Flash, we used carefully designed, domain-specific prompts (Multimedia Appendix 1). These prompts enforced specific constraints: they defined clinical context (eg, diagnosis type), removed noise, and ensured that terms were translated strictly into the official medical vocabulary. However, the accuracy of subsequent mapping is entirely dependent on translation quality. Despite these controlled prompts, we currently lack external gold-standard metrics or formal error analysis for this critical phase.

We note that the volume of local terms requiring Korean translation was low, at approximately 1.5% of the total mapped terms. While this proportion is small, we acknowledge that translation inaccuracies in even a limited subset of terms could introduce minor bias into the overall mapping accuracy. Accordingly, we recognize the necessity of integrating translation-specific metrics into the overall evaluation framework.

Finally, while the gold standard for mapping underwent extensive review by experienced terminology experts both internally and externally, we did not collect or report an objective measure of interrater agreement, such as Cohen κ. Although this expert involvement likely improved reliability, the absence of a quantitative assessment remains a limitation and should be considered when interpreting our results.

### Future Directions

Although retrieval-augmented generation was explored in our preliminary model, we observed limitations where the current general-purpose LLM failed to accurately leverage the inherent ontological structure of SNOMED CT, often yielding results identical to simple vector similarity search [34]. Therefore, further improvement is expected by using LLMs that are specifically designed to leverage both contextual information and the intrinsic structure of SNOMED CT in future research. Further, the use of SNOMED CT’s ontology structure and knowledge graphs (eg, GraphRAG) is expected to enhance semantic retrieval and disambiguation capabilities, increasing accuracy and robustness across broader domains [35]. To improve authoring of new concepts, automation features applying detailed MRCM cardinality rules and an advanced inactivation process will be developed, reducing modeling errors and ensuring SNOMED CT compliance.

From a sustainability perspective, widespread adoption will require continued resource investment for tool maintenance and updates, as well as collaboration across clinicians, informaticians, terminology experts, and policymakers. Keeping standard codes current, ensuring ongoing quality assurance, and adapting to multilingual and ICD-11 (International Classification of Diseases, 11th Revision) or International Classification of Health Interventions-integrated environments—particularly for mapping complex surgical procedures—are essential for scalable and future-proof deployment. Despite concerns about the potential for increased operational costs when deploying commercial LLMs, our experimental results indicate that these expenses were negligible relative to the benefits achieved. For example, embedding 1.6 million tokens costs just US $0.03, and translating 1779 cases incurred only minimal costs based on standard token pricing. These findings highlight the economic viability and sustainability of incorporating LLM components into automated pipelines.

Our long-term vision is to deliver this solution as a cloud-based software-as-a-service platform, supporting agile terminology standardization across diverse medical institutions both domestically and worldwide. For effective adoption in new environments, local verification and adaptation may be necessary to address differences in data standards and clinical practices. Notably, the system’s methodological approach aligns with the World Health Organization’s and Centers for Disease Control and Prevention’s international guidelines, supporting its potential generalizability across health systems worldwide [11,12].

### Conclusions

We developed and validated the first large-scale, multi-institutional automatic terminology mapping tool supporting both pre- and postcoordinated SNOMED CT concepts. The tool substantially reduces manual workload, improves mapping accuracy, and enhances interoperability for clinical data across the evaluated sites. While our current evaluation was limited to internal implementation, further work will focus on advancing commercialization and expanding deployment. We anticipate that, with continued refinement and local verification, this tool could support terminology standardization and interoperability for broader national and international healthcare data integration in the future.

## Data Availability

The datasets generated and analyzed during this study are not publicly available due to the hospital policies.

## Appendix

Multimedia Appendix 1 GPT-4o translation prompt.

## Abbreviations

AI: artificial intelligence
API: application programming interface
DSMC: DongSan Medical Center
EUMC: Ewha Womans University Medical Center
FSN: fully specified name
HRS: Healthcare Data Research Suite
ICD-11: International Classification of Diseases, 11th Revision
IRB: institutional review board
KHMC: KyungHee University Medical Center
KUMC: Korea University Medical Center
LLM: large language model
MRCM: Machine Readable Concept Model
OWL: Web Ontology Language
SNOMED: Systematized Nomenclature of Medicine
SNOMED CT: Systematized Nomenclature of Medicine Clinical Terms
UI: user interface
